# High Prevalence of Shared International Type 53 among *Mycobacterium tuberculosis* Complex Strains in Retreated Patients from Côte d’Ivoire

**DOI:** 10.1371/journal.pone.0045363

**Published:** 2012-09-18

**Authors:** Timothée Ouassa, Emanuele Borroni, Guillaume Yao Loukou, Hortense Faye-Kette, Jacquemin Kouakou, Hervé Menan, Daniela Maria Cirillo

**Affiliations:** 1 Department of Bacteriology and Virology, Faculty of Pharmacy, University of Cocody, Abidjan, Côte d’Ivoire; 2 Centre for Diagnosis and Research on AIDS and opportunistic infections, Teaching Hospital of Treichville, Abidjan, Côte d’Ivoire; 3 Emerging Bacterial Pathogen Unit, San Raffaele Scientific Institute, Milan, Italy; 4 Department of Bacteriology and Virology, Faculty of Medicine, University of Cocody, Abidjan, Côte d’Ivoire; 5 National Tuberculosis Program, Abidjan, Côte d’Ivoire; 6 Centre for Diagnosis and Research on AIDS and opportunistic infections, Teaching Hospital of Treichville, Abidjan, Côte d’Ivoire; University of Padova, Italy

## Abstract

**Background:**

Genotyping methods are useful tools to provide information on tuberculosis epidemic. They can allow a better response from health authorities and the implementation of measures for tuberculosis control. This study aimed to identify the main lineages and clades of *Mycobacterium tuberculosis* complex strains circulating in Côte d’Ivoire.

**Methods/Main Findings:**

Strains isolated from sputum samples of patients ongoing retreatment from all the country were characterized by spoligotyping and by MIRU-VNTR. Profiles obtained by spoligotyping were first compared to the SITVIT/SpolDB4 database for family assignment. Of 194 strains analysed, 146 (75.3%) belonged to the T lineage. The most predominant spoligotype was the shared international type 53 with 135 strains (69.6%). In contrast with neighbouring countries, LAM (11 strains, 5.7%) and H (9 strains 4.6%) lineages were slightly represented. Only 3 Beijing strains (1.5%) and 4 strains of *Mycobacterium africanum* (2%) were found. Analysis of the results obtained with MIRU-VNTR revealed also a high level of clustering.

**Conclusion/Significance:**

The population of *Mycobacterium tuberculosis* complex strains among retreatment cases in Côte d’Ivoire exhibits a low diversity, allowing to assume recent transmission and locally based infection.

## Introduction

Côte d'Ivoire is one of the countries with highest tuberculosis (TB) incidence in the world with an estimated incidence rate of 139 cases per 100 000 inhabitants [1]. This situation is worsened by the HIV/AIDS epidemics as Côte d’Ivoire is also one of the most affected countries in Africa with an estimated prevalence of 3.4% in the whole population [2] and 24% in TB patients [1].

Measures taken to control the infection should go through the early detection and treatment of cases but also, knowledge of genetic structure of *Mycobacterium tuberculosis* complex (MTBC) population in the country. Indeed, efforts in TB control and prevention meanly rely on recommendations to stop TB transmission and little information is available on molecular epidemiology of TB in Côte d’Ivoire.

However, molecular genotyping is very useful for understanding TB epidemiology. Many tools are currently available for this purpose: IS6110 RFLP [3], [4], spoligotyping [5], MIRU-VNTR [6], [7], SNPs [8]–[10], LSPs [11], [12] and among these methods, spoligotyping which is a PCR-based technique has the advantage of being inexpensive and reproducible [13]. It is also the one to be firstly recommended for identifying the main lineages and clades in a given country. It could thereafter be combined with other more discriminative methods for transmission studies or strain differentiation at the clonal level [14], [15].

It should be noted that very few molecular studies have been conducted to date in Côte d’Ivoire. One of them has included 15 strains isolated from new cases and in one region of the country [16].The limited number of strains tested could not, however, reflect the actual situation in the country. Thus, there is a need for conducting a study on a larger scale, involving a larger number of strains.

In this study, we characterized by spoligotyping and MIRU-VNTR, MTBC strains during the monitoring of retreated patients in Côte d’Ivoire over a 1-year period (2008–2009) in order to assess their genetic diversity.

## Materials and Methods

### Bacterial Strains

The national TB policy is currently limited to tuberculosis screening and treatment of smear-positive patients. Culture of sputum is not yet systematic, mainly for cost reasons, but also for the lack of structures in which it could be implemented.

**Figure 1:**
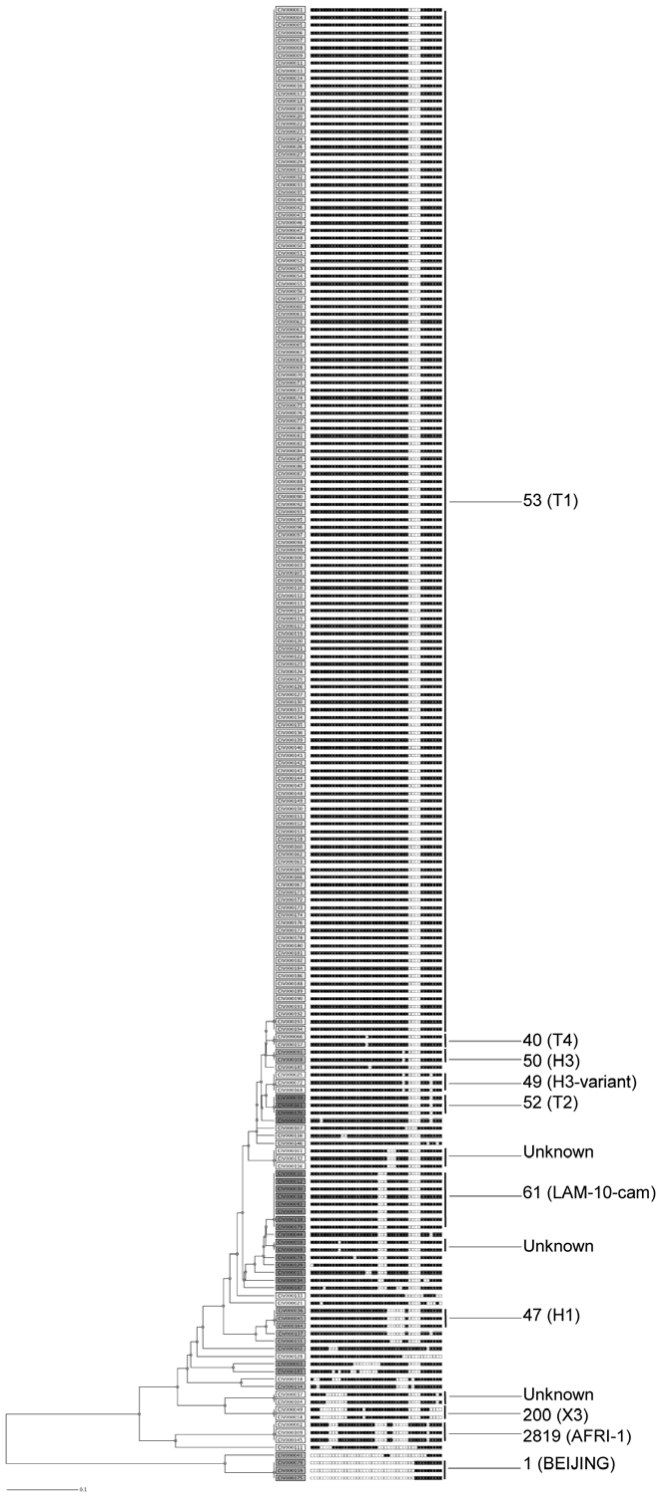
UPGMA type dendrogram generated using spoligotypes profiles on the MIRU-VNTRplus website. Thirteen clusters were observed. SIT numbers of clusters are indicated with corresponding clades in brackets.

**Table 1:** Spoligotypes identified by a SIT number in the SITVIT database.

SIT^1^	Spoligotype Description^2^	Octal code	Number (%)	Clade^3^
1	□□□□□□□□□□□□□□□□□□□□□□□□□□□□□□□□□□▪▪▪▪▪▪▪▪▪	000000000003771	3 (1.5)	Beijing
40	▪▪▪▪▪▪▪▪▪▪▪▪▪▪▪▪▪▪□▪▪▪▪▪▪▪▪▪▪▪▪▪□□□□▪▪▪▪▪▪▪	777777377760771	2 (1.0)	T4
47	▪▪▪▪▪▪▪▪▪▪▪▪▪▪▪▪▪▪▪▪▪▪▪▪▪□□□□□□▪□□□□▪▪▪▪▪▪▪	777777774020771	3 (1.5)	H1
49	▪▪▪▪▪▪▪▪▪▪▪▪▪▪▪▪▪▪▪▪▪▪▪▪▪▪▪▪▪▪□▪□□□□▪▪▪□▪▪▪	777777777720731	3 (1.5)	H3-variant
50	▪▪▪▪▪▪▪▪▪▪▪▪▪▪▪▪▪▪▪▪▪▪▪▪▪▪▪▪▪▪□▪□□□□▪▪▪▪▪▪▪	777777777720771	2 (1)	H3
52	▪▪▪▪▪▪▪▪▪▪▪▪▪▪▪▪▪▪▪▪▪▪▪▪▪▪▪▪▪▪▪▪□□□□▪▪▪□▪▪▪	777777777760731	3 (1.5)	T2
53	▪▪▪▪▪▪▪▪▪▪▪▪▪▪▪▪▪▪▪▪▪▪▪▪▪▪▪▪▪▪▪▪□□□□▪▪▪▪▪▪▪	777777777760771	135 (69.6)	T1
57	▪▪▪▪▪▪▪▪▪▪▪▪▪▪▪▪▪▪□□▪▪□□□▪▪▪▪▪▪▪□□□□▪▪▪▪▪▪▪	777777143760771	1 (0.5)	LAM10-CAM
61	▪▪▪▪▪▪▪▪▪▪▪▪▪▪▪▪▪▪▪▪▪▪□□□▪▪▪▪▪▪▪□□□□▪▪▪▪▪▪▪	777777743760771	8 (4.1)	LAM10-CAM
62	▪▪▪▪▪▪▪▪▪▪▪▪▪▪▪▪▪▪▪▪▪▪▪▪▪□□□□□□▪□□□□▪▪▪□▪▪▪	777777774020731	1 (0.5)	H1
115	▪▪▪▪▪▪▪▪▪▪▪▪▪▪□▪▪▪▪▪▪▪□□□▪▪▪▪▪▪▪□□□□▪▪▪▪▪▪▪	777767743760771	1 (0.5)	LAM10-CAM
120	▪▪▪▪▪▪▪▪▪▪▪▪▪▪▪▪▪▪▪□▪▪▪▪▪▪▪▪▪▪▪▪□□□□▪▪▪▪▪▪▪	777777577760771	1 (0.5)	T1
181	▪▪▪▪▪▪□□□▪▪▪▪▪▪▪▪▪▪▪▪▪▪▪▪▪▪▪▪▪▪▪▪▪▪▪▪▪□▪▪▪▪	770777777777671	1 (0.5)	AFRI1
200	▪▪▪□□□□□□□□□▪▪▪▪▪□▪▪▪▪▪▪▪▪▪▪▪▪▪▪□□□□▪▪▪□□□□	700076777760700	2 (1)	X3
203	▪▪▪□□□□▪▪▪▪▪▪▪▪▪▪▪▪▪▪▪□□□□□□□□□□□□□▪▪▪▪▪▪▪▪	703777740001771	1 (0.5)	CAS
237	▪▪▪▪▪▪▪▪▪▪▪▪▪▪▪▪▪▪▪▪▪▪▪▪▪▪▪▪▪▪□□□□□□□□□□□□□	777777777700000	1 (0.5)	U (likely H3)
340	▪□□▪▪▪▪□□□▪▪▪▪▪▪▪▪▪▪▪▪▪▪▪▪▪▪□□□□▪□▪▪▪▪▪▪▪▪▪	474377777413771	1 (0.5)	EAI5
342	▪▪□▪▪▪▪▪▪▪▪▪▪▪▪▪▪▪▪▪▪▪▪▪▪▪▪▪□□□□▪□▪▪▪▪▪▪▪▪▪	677777777413771	1 (0.5)	EAI5
370	▪▪▪▪▪▪▪▪▪▪▪▪▪▪▪▪▪▪▪▪▪▪□□▪▪▪▪▪▪▪▪□□□□▪□□▪▪▪▪	777777747760471	1 (0.5)	T1
403	▪▪▪▪▪▪▪▪▪▪▪▪▪▪▪▪▪▪▪▪▪▪□□□▪▪▪▪▪▪▪□□□□▪▪▪□▪▪▪	777777743760731	1 (0.5)	LAM10-CAM
551	▪▪▪□▪▪▪▪▪▪▪▪▪▪▪▪▪▪▪▪▪▪▪▪▪▪▪▪▪▪▪▪□□□□▪▪□□□▪□	737777777760610	1 (0.5)	T1
848	▪▪▪□▪▪▪▪▪▪▪▪▪▪▪▪▪▪▪▪▪▪▪▪▪▪▪▪▪▪▪▪□□□□▪▪▪□▪▪▪	737777777760731	1 (0.5)	T2
1056	▪▪▪▪▪▪▪▪▪▪□□▪▪▪▪▪▪▪▪▪▪▪▪▪▪▪▪▪▪▪▪□□□□▪▪▪▪▪▪▪	777477777760771	1 (0.5)	T2
1737	▪▪▪▪▪▪▪▪▪▪▪▪▪▪□□□□□□□□□▪▪▪▪▪▪▪▪▪□□□□▪▪▪▪▪▪▪	777760017760771	1 (0.5)	T1

1 SIT: Shared International Type.

2 The black and white boxes indicate the presence and absence, respectively, of the specific spacer at positions 1–43 in the DR locus.

3 Clade designations according to SpolDB4 database.

**Table 2:** Spoligotypes of orphan strains and clusters not identified in the SITVIT database.

Strain^1^	Spoligotype description^2^	Octal code	Clustered/not clustered
CIV000107	▪▪▪▪▪▪▪▪▪▪▪▪▪▪▪▪▪▪▪▪▪▪▪▪▪▪▪▪▪▪▪□□□□▪▪▪▪▪▪▪▪	777777777741771	Not clustered
CIV000133	▪▪▪▪▪▪▪▪▪▪▪▪▪▪▪▪▪▪▪▪▪▪▪▪▪▪▪▪▪▪▪□□□□□□▪▪▪▪□□	777777777740360	Not clustered
CIV000146	▪▪▪▪▪▪▪▪▪▪▪▪▪▪▪▪▪▪▪▪▪▪▪▪▪▪▪▪▪▪▪▪□□□□▪▪□▪▪□▪	777777777760661	Not clustered
CIV000157	▪▪▪▪▪▪▪▪▪▪▪▪▪▪▪▪▪▪▪▪▪▪▪▪▪▪□□□□▪▪□□□□▪▪▪▪▪▪▪	777777776060771	Not clustered
CIV000101	▪▪▪▪▪▪▪▪▪▪▪▪▪▪▪▪▪▪▪▪▪▪▪▪▪□□□▪▪▪▪□□□□▪▪▪▪▪▪▪	777777774360771	Clustered
CIV000132	▪▪▪▪▪▪▪▪▪▪▪▪▪▪▪▪▪▪▪▪▪▪▪▪▪□□□▪▪▪▪□□□□▪▪▪▪▪▪▪	777777774360771	Clustered
CIV000156	▪▪▪▪▪▪▪▪▪▪▪▪▪▪▪▪▪▪▪▪▪▪▪▪▪□□□▪▪▪▪□□□□▪▪▪▪▪▪▪	777777774360771	Clustered
CIV000059	▪▪▪▪▪▪▪▪▪□▪▪▪▪▪▪▪▪▪▪▪▪□□□▪▪▪▪▪▪▪□□□□▪▪▪▪▪▪▪	777377743760771	Clustered
CIV000169	▪▪▪▪▪▪▪▪▪□▪▪▪▪▪▪▪▪▪▪▪▪□□□▪▪▪▪▪▪▪□□□□▪▪▪▪▪▪▪	777377743760771	Clustered
CIV000183	▪▪▪▪▪▪▪□▪□▪▪▪▪□□▪□□□□□□▪▪▪▪▪▪▪▪▪□□□□▪▪▪▪▪▪▪	775344037760771	Not clustered
CIV000037	▪▪▪▪▪□□□□□□□▪▪▪▪▪▪▪▪▪▪▪▪▪▪▪▪▪▪▪▪□□□□▪▪▪▪▪□▪	760077777760761	Clustered
CIV000104	▪▪▪▪▪□□□□□□□▪▪▪▪▪▪▪▪▪▪▪▪▪▪▪▪▪▪▪▪□□□□▪▪▪▪▪□▪	760077777760761	Clustered
CIV000187	▪▪▪▪□▪▪▪▪▪▪▪▪▪▪▪▪▪▪▪▪▪□□□▪▪▪□□▪▪□□□□▪▪▪▪▪▪▪	757777743460771	Not clustered
CIV000129	□▪▪▪▪▪▪▪▪▪▪▪▪▪▪▪▪▪▪▪▪▪▪□□□▪▪▪▪▪▪□□□□▪▪▪▪▪▪▪	377777743760771	Not clustered
CIV000041	□□□□□□□□□□□□□□□□□□□□□□□□▪▪□□□□□□□□□□□□▪▪▪▪▪	000000006000171	Not clustered
CIV000002	▪▪▪▪▪▪□□□▪▪▪▪▪▪▪▪▪▪▪▪□□□□▪▪□□□□□▪▪▪▪▪▪□▪▪▪▪	770777703017671	Clustered
CIV000109	▪▪▪▪▪▪□□□▪▪▪▪▪▪▪▪▪▪▪▪□□□□▪▪□□□□□▪▪▪▪▪▪□▪▪▪▪	770777703017671	Clustered
CIV000145	▪▪▪▪▪▪□□□▪▪▪▪▪▪▪▪▪▪▪▪□□□□▪▪□□□□□▪▪▪▪▪▪□▪▪▪▪	770777703017671	Clustered

1 DNA identification in the sample database.

2 The black and white boxes indicate the presence and absence, respectively, of the specific spacer at positions 1–43 in the DR locus.

Within the framework of a decision to monitor drug resistance in patients undergoing retreatment (i.e. failure, relapse or recurrence or default), sputum samples from the 16 TB centres located throughout the country and from the main pneumophtisiology departments were collected. This study was conducted on MTBC strains isolated during a 1-year period from December 2008 to December 2009.

**Figure 2:**
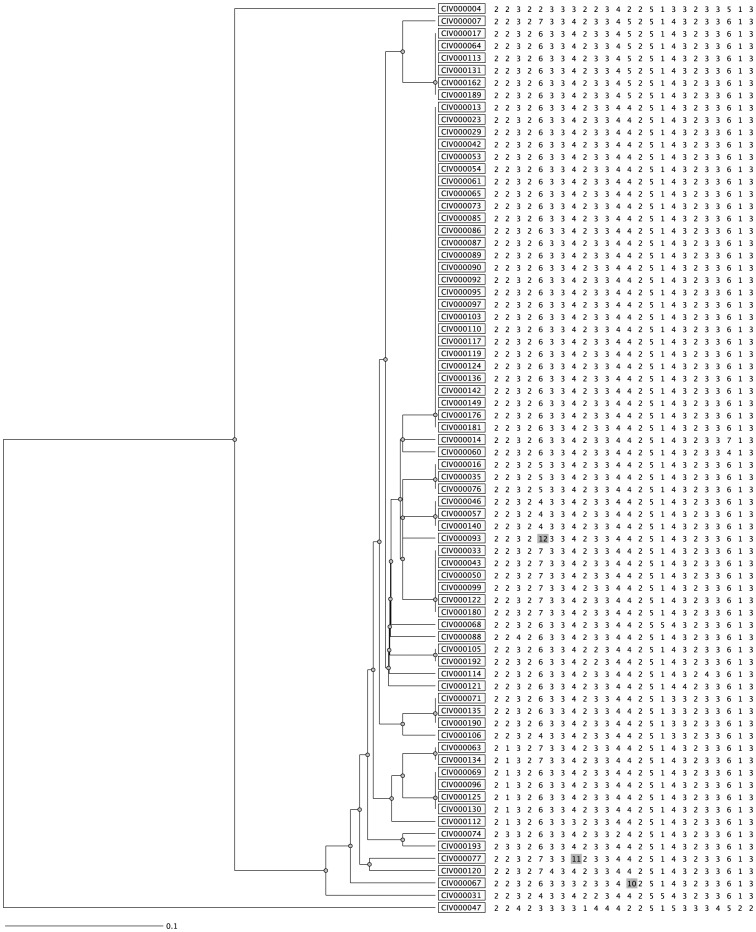
Dendrogram generated using MIRU-VNTR profiles of 74 strains identified as SIT 53 by spoligotyping. Samples CIV000067, CIV000077 and CIV000093 are characterized by the presence of double alleles for Mtub30 (4+2), Mtub21 (4+3) and Miru40 (5+2) respectively.

### Drug Resistance Detection

Detection of susceptibility to rifampicin and isoniazid was made using the GenoType MTBDR*plus* (Hain Lifescience, Nehren, Germany).

**Table 3:** Drug resistance patterns according to lineages.

Lineages	Drug resistance profile^1^				
	Susceptible	Rif R	Inh R	MDR	Total
AFRI	1 (25.0)	0	0	3 (75.0)	4
Beijing	1 (33.3)	1 (33.3)	1 (33.3)	0	3
CAS	0	0	0	1	1
EAI	1 (50.0)	0	1 (50.0)	0	2
H	6 (85.7)	0	0	1 (14.3)	7
Lam	5 (45.5)	4 (36.4)	0	2 (18.2)	11
S	0	0	0	2 (100)	2
T	7 (5.0)	0	6 (4.2)	128 (90.8)	141
U	0	0	0	1 (100)	1
Unknown	6 (85.7)	0	0	1 (14.3)	7
X	2 (100)	0	0	0	2
Total	29 (16.0)	5 (2.8)	8 (4.4)	139 (76.8)	181

1 Percentages are in brackets.

The method was performed as described by the manufacturer. Briefly, the reaction was performed in 2 steps. For amplification, 5 µl of DNA extract were added to 35 µl of primers and nucleotides (provided by the manufacturer), 10 µl of 10× amplification buffer, 1.2 µl of a solution of 2,5 mM MgCl2, 1.25 U Hot Start Taq DNA polymerase (Qiagen GmbH, Hilden, Germany) and water to a final volume of 50 µl. The hybridization step took place in a hybridization buffer at 45°C. The revelation of the hybridization was made after a stringent washing by colorimetric reaction.

After amplification, hybridization and detection were performed in an automated washing, shaking and heating machine, the GT-Blot 20 (Hain Lifescience GmbH, Nehren, Germany).

### Spoligotyping

Spoligotyping was performed on bacterial strains as described by Kamerbeek et al. [5] using a commercially available kit (Isogen Life Science B.V., Utrecht, The Netherlands). Manipulations were performed according to the manufacturer’s recommendations.

### MIRU-VNTR

Amplification of loci was performed by using the 24 loci MIRU-VNTR typing kit (Genoscreen, Lille, France).

Briefly, 8 µl of each of the 8 ready-to-use multiplex premixes was dispensed in the 12 wells of the lines of a 96-wells plate. Further, 2 µl of DNA was added in each of the 8 wells of a row for each of the 12 samples tested by plate. In each plate, was included a positive control consisting in DNA from a strain of MTBC H37Rv.

Automated MIRU-VNTR analysis was performed as previously described by Supply et al [17], with a few modifications. For each multiplex PCR, 2 µl of PCR products was added to 10 µl of a loading buffer containing 9.5 µl of Hi-Di and 0.5 µl of Gene Scan 1200 LIZ size standard (Applied Biosystems, California, USA). Before being loaded, samples were denatured at 95°C for 5 min and then kept on ice. The samples were then analyzed by using an automatic sequencer, the ABI 3730 DNA analyser (Applied Biosystems, California, USA),

Estimation of the sizes of the PCR fragments was done using the Genomapper v3.7 software (Applied Biosystems, California, USA) with automated assignment of each alleles.

### Family Assignment

Identification of spoligotypes was done by using the international SpolDB4/SITVIT database [18] available at http://www.pasteur-guadeloupe.fr:8081/SITVITDemo/and also the freely assessable MIRU-VNTR*plus* website [19], [20]. Definition of families and lineages was done by comparing the observed profiles with those contained in databases. The Spotclust database [21] available at http://tbinsight.cs.rpi.edu/run_spotclust.html was used for spoligotype patterns not previously reported in SpolDB4.

### Ethical Considerations

All the mycobacterial strains analyzed in this study were available as a part of regular activities of the National Tuberculosis Program (NTP) of Côte d’Ivoire. Patients were managed according to NTP guidelines and the Centre for Diagnosis and Research on aids and opportunistic infections (CeDReS), which is a reference laboratory for the NTP routinely stores isolated bacterial strains for disease surveillance.

The current study was descriptive of a bacterial collection and contained no material of human origin. Ethical approval was therefore not required. Also, there was no direct contact with the patients, the outcome of the research data would not affect the patient management and data were analyzed anonymously. For the same reason, patients’ consent was not required.

## Results

### Drug Resistance

Thirteen strains gave uninterpretable results with of the Genotype MTBDR*plus*. For the 181 remaining strains, 29 (16%) were susceptible to both Rifampicin and Isoniazid while 139 (76,8%) were multidrug resistant (MDR).

### Spoligotyping

A total of 194 strains were analyzed and yielded 37 spoligotypes (Figure 1). One hundred seventy-six strains (90.7%) representing 24 profiles were already known in the SITVIT database and had a shared international type number (SIT) (Table 1). Fifteen of these profiles were unique and 161 were grouped into 9 clusters, the biggest one containing alone 135 strains. The remaining 18 strains (9.3%) were not identified yet in the SITVIT database (Table 2). Of these, 8 were unique while 10 were grouped into four clusters; two of 3 strains each and two of 2 strains each.

Irrespective of the database comparison, 13 spoligotypes corresponded to clusters, amounting to an overall clustering rate of 88.1% (171/194).

Classification by principal genetic groups (PGGs) gave for the PGG1 (ancient lineages) only 10 strains (5.2%) and 184 strains (94.8%) for PGG2/3 (modern lineages).

The most prevalent was the T lineage with 146 strains (75.3%). The predominance of the ubiquitous SIT53 is noteworthy as 135 strains (69.6%) corresponded to that single spoligotype. The other lineages accounted for a smaller number of strains. It was the case for LAM with 11 strains (5.7%) and H with 9 strains (4.6%). The Beijing lineage was very poorly represented with only 3 strains (1.5%). For the X and CAS lineages, they were 2 strains (1.0%) and one single strain (0.5%) respectively. Additionally to a strain identified as *Mycobacterium africanum* in SpolDB4 database, one unidentified cluster of 3 strains with the octal code 770777703017671 was defined as *Mycobacterium africanum* by Spotclust and thereafter found to belong to *Mycobacterium africanum* type 2 clade as observed by de Jong et al. [22]. Finally, four strains of *Mycobacterium africanum* (2%) were present while no strain of *Mycobacterium bovis* was part of the MTBC strains studied.

### MIRU-VNTR

Seventy four samples identified as SIT 53 by spoligotyping were analysis and lead to 23 profiles. Sixteen of these were unique while most of the samples (58/74, 78;4%) were clustered into 9 profiles (Figure 2).

In 3 strains (4%), the presence of double alleles was observed but for only one locus in each case.

### Drug Resistance and Lineages

In lineages containing more than one strain, higher proportion of MDR strains was found in S, T and Africanum families with 100% (2/2), 90.8% (128/141) and 75% (3/4) respectively (Table 3). However, no MDR strains were found in Beijing and X families, and the rates was quite low in H and Lam families with 14.3% (1/7) and 18.2% (2/11) respectively.

## Discussion

This study reports the first use of spoligotyping and 24 loci MIRU-VNTR at a large scale to assess the genetic diversity of MTBC strains circulating in Côte d’Ivoire. The main comment on the results is the clear predominance of the T lineage, and particularly SIT53 which reflects a relative homogeneity of MTBC strains isolated in Côte d’Ivoire. This observation, in comparison with studies in neighbouring West African countries shows a great difference. Indeed, prevalence of T lineage strains were 21.7%, 19.9%, 10.3%, 5.7% and 4% in Burkina Faso, Ghana, Sierra Leone, Benin and Nigeria respectively [23]–[27].

The very high prevalence of T lineage in general and of ubiquitous SIT53 in particular makes Côte d’Ivoire an exception within the West African sub-region and even to the extent in Africa and in the world when considering strains repartition in the SITVIT database. One explanation could be the characteristics of the population studied. Indeed, in most other countries, data resulted from drug susceptibility surveillance studies in new cases [23], [24], [26], [27]. In this study, however, the fact that patients were undergoing a retreatment regimen could be the source of selection bias. Indeed, in Sierra Leone, a study conducted also in retreatment cases led to very different results [23], showing a high genetic diversity among MTBC strains. However, one great difference in this study was that lower number of MDR strains was included.

Another cause can be the fact that SIT53 spoligotype had sometimes been associated with mixed infection as observed in a study conducted in South Africa, a country characterized by a high prevalence of TB [28]. Lazzarini et al. also hypothesized that as SIT53 has a high risk to result from a combination of different spoligotype patterns and that many of the SIT53 spoligotypes could be in fact the result of mixed infections [29].

In this study, however, only 3 double allele cases were detected by MIRU-VNTR. They were observed for one single locus in each case, suggesting the presence of an allelic variant rather than mixed infection cases.

Eventually, despite all these hypotheses, it is useful to mention that a 12 years old study can support the findings of this study. Indeed, the authors reported that the same spoligotype profile was observed in 55% (11/20) of MTBC strains from Côte d’Ivoire when only 10 out of the 69 strains from Senegal (14.5%) had this profile [30].

As a consequence, and in contrast to other West African countries, other lineages are underrepresented in Côte d’Ivoire. Indeed, the LAM lineage and particularly the SIT61 belonging to the LAM10-CAM clade accounted for 59.5%, 30%, 25.3%, 20.6% and 15.5% of the cases, respectively in Nigeria, Ghana, Burkina Faso, Benin and in Sierra Leone [23]–[27] but for only 5.7% in Côte d’Ivoire.

About the worldly represented Beijing lineage, its prevalence in the study was relatively low. The same observation was made in Ghana and Sierra Leone with 4% of prevalence each [24], [25]. No Beijing strains were observed in Burkina Faso or in Nigeria [23], [27]. However, the prevalence (20/194, 10.3%) was relatively high in Benin [26] comparatively to other countries of the region.

Finally, it was the same situation for *Mycobacterium africanum* which is supposed to be predominant in West Africa with an epicentre in Guinea-Bissau [22], [31], [32]. This fact is demonstrated by the high prevalence of this specie not only in Guinea-Bissau but also in Ghana, in Sierra Leone and in the Gambia with 20%, 24%, and 38% respectively [24], [25], [22]. However, despite its high proportion among strains from Côte d’Ivoire in the SITVIT database, *Mycobacterium africanum* was slightly represented in our study.

The use of MIRU-VNTR to assess genotypic diversity among bacterial strains belonging to SIT 53, the biggest cluster obtained by spoligotyping VNTR led to a better discrimination but still revealed a high level of clustering.

Côte d’Ivoire seems therefore to be a country where strains of *Mycobacterium tuberculosis* complex exhibit a certain consistency which can raise suspicion for recent and locally based transmission even if in this case, all strains were isolated from retreated patients. These findings should, however be confirmed by larger studies including new TB cases.
